# Construction and Immunogenicity of a Recombinant Pseudorabies Virus Expressing the Major Neutralizing Epitopes A and D of Transmissible Gastroenteritis Virus Spike Protein

**DOI:** 10.3390/vetsci13090914

**Published:** 2026-09-05

**Authors:** Li Zhao, Xiang-Shuo Tian, Tong Xu, Xiao-Zhan Zhang, Ying-Hui Wen, Xing-Hui Song, Zhong-Yi Fang, Yi-Lei Li, Hong-Ying Chen

**Affiliations:** 1 College of Veterinary Medicine, Henan University of Animal Husbandry and Economy, Zhengdong New District Longzi Lake 15#, Zhengzhou 450046, China; 2 College of Veterinary Medicine, Henan Agricultural University, Zhengdong New District Longzi Lake 15#, Zhengzhou 450046, China; 3 Henan Institute of Agricultural and Animal Product Inspection and Technology, Zhengzhou 450008, China

**Keywords:** pseudorabies virus, transmissible gastroenteritis virus, neutralizing epitope, CRISPR/Cas9, recombinant vaccine

## Abstract

Transmissible gastroenteritis (TGE) and pseudorabies (PR) remain important viral diseases causing massive economic losses and posing a continuous burden on the global swine industry. The continuous emergence of variant strains of transmissible gastroenteritis virus (TGEV) and pseudorabies virus (PRV) has gradually weakened the protective efficacy of traditional vaccines, highlighting the urgent need for next-generation preventive vaccine candidates. Here, we constructed a recombinant pseudorabies virus named rPRV-AD expressing the major neutralizing epitopes A and D of the TGEV spike protein via homologous recombination combined with CRISPR/Cas9-gene editing technology and then evaluated its biological characteristics in vitro and immunogenicity in piglets. The results showed that this recombinant virus exhibited similar replication kinetics and biological properties to the parental strain. Immunization of 2-week-old piglets with rPRV-AD caused no obvious adverse effects and induced specific antibody and neutralizing antibody responses against both TGEV and PRV. Following virulent TGEV challenge, compared with the DMEM control group, rPRV-AD immunization alleviated clinical signs of piglets and significantly reduced viral load in intestine and feces, although its protective efficacy was lower than that of the commercial TGEV vaccine. Moreover, rPRV-AD provided effective clinical protection against challenge with the virulent PRV NY strain. In summary, these findings suggest that rPRV-AD represents a vaccine candidate that provides partial protection and warrants further optimization, and yet shows short-term protective efficacy against both PRV and TGEV in pigs.

## Simple Summary

Transmissible gastroenteritis and pseudorabies are two viral diseases that severely affect pig farms, causing substantial economic losses. Conventional vaccines have shown declining protective efficacy. We built a recombinant virus called rPRV-AD that carries key bits of the transmissible gastroenteritis virus (TGEV) spike protein. In the lab, it grew just like the original parent virus. When administered to 2-week-old piglets in this preliminary animal trial, the vaccinated animals remained clinically healthy and produced antibodies against both viruses. After virus challenge, vaccinated pigs exhibited milder clinical signs and reduced viral loads in intestines and feces. Under our experimental conditions, rPRV-AD exhibited lower protective efficacy against TGEV than a commercial vaccine, while comparable protective observations were obtained against pseudorabies.

## 1. Introduction

Transmissible gastroenteritis (TGE) is a severe enteric disease of swine manifested by vomiting, severe diarrhea, and dehydration, with mortality rates particularly high in neonatal piglets under two weeks of age. First identified in the United States in 1946, it has since been reported in many countries across the Americas, Asia, and Europe, resulting in considerable economic losses for the global swine industry [1,2]. Transmissible gastroenteritis virus (TGEV), the etiological agent of TGE, is an enveloped single-stranded positive-sense RNA virus belonging to the genus *Alphacoronavirus* within the family *Coronaviridae*. The TGEV genome is about 28.5 kb and contains nine ORFs. ORF1a and ORF1b encode polyproteins that are processed into 16 non-structural proteins (NSP1–16) involved in viral replication. The remaining ORFs code for four structural proteins—spike (S), envelope (E), membrane (M), and nucleocapsid (N)—and three accessory proteins (ORF3a, ORF3b, and ORF7 [3]. The S protein, containing 1447 amino acids (aa), mediates host–cell attachment and entry by interacting with porcine aminopeptidase N and sialic acids, which serve as cellular receptor and attachment factor respectively. Beyond facilitating viral entry, the S protein functions as the key immunogen responsible for eliciting virus-neutralizing antibodies [4,5]. Four major antigenic sites, termed A, B, C and D, have been mapped to the N-terminal domain of the S protein. Sites A and D are regarded as the dominant neutralizing epitopes, which makes them highly desirable targets for developing TGEV-based vaccines [6,7,8].

Pseudorabies virus (PRV), a member of the subfamily *Alphaherpesvirinae*, causes multisystemic disease in pigs across all age groups. Clinical outcomes of PRV infection vary considerably: neonatal piglets may develop fatal encephalitis; growing-finishing pigs exhibit respiratory distress and growth retardation; pregnant sows suffer reproductive failure including abortion and stillbirth. Taken together, these manifestations impose a substantial economic burden on the global swine industry [9,10,11]. The PRV genome is a linear double-stranded DNA molecule of approximately 143 kb, consisting of a unique long region [12], a unique short (US) region, and internal and terminal repeat sequences (IRS and TRS, respectively) [13]. Several genes within the PRV genome are dispensable for viral replication, including thymidine kinase (TK), glycoprotein E (gE), glycoprotein I (gI), and glycoprotein G (gG). Deletion or replacement of these non-essential genes with foreign antigen-expression cassettes barely impairs viral replication capacity, making PRV a flexible vector to deliver antigens from other swine pathogens. Accordingly, attenuated PRV strains have been extensively evaluated as viral vectors to develop multivalent vaccines against pseudorabies and other economically important swine diseases [14,15]. The CRISPR/Cas9 gene-editing system has further enhanced the efficiency and accuracy of recombinant PRV generation, accelerating the development of new vaccine candidates [16,17,18]. Virulence reversion and viral shedding are prominent risks of conventional attenuated vaccines; however, multi-gene-deleted attenuated live-vector vaccines carrying stable virulence-gene deletions can effectively reduce these risks. In this study, the AD fragment harbouring major neutralizing epitopes A and D from the TGEV spike protein was inserted into the gG locus of the PRV genome. The resulting recombinant virus was designated rPRV-AD. This construct was designed to serve as a bivalent vaccine, capable of eliciting effective dual-specific immune protection against both PRV and TGEV in pigs.

Vaccination remains the cornerstone for controlling TGEV and PRV infections. Recombinant PRV vectors expressing heterologous protective antigens represent a promising platform for developing multivalent vaccines against economically important swine diseases [19,20]. However, antigenically and genetically divergent PRV variants have circulated since 2011 among pig herds immunized with the Bartha-K61 vaccine in China, greatly reducing the protective efficacy of the classical Bartha-K61 vaccine [9,11]. To address this issue, several attenuated vaccine candidates based on prevailing PRV field isolates, such as JS-2012-ΔgE/gI and NY-gE^−^/gI^−^/TK^−^, have been generated and preliminarily assessed by researchers [14,21,22,23]. In this study, we generated a recombinant virus rPRV-AD expressing the major neutralizing epitopes A and D from the TGEV S protein using homologous recombination combined with CRISPR/Cas9 gene editing, and systematically characterized its in vitro properties, genetic stability, safety, and protective efficacy against virulent TGEV and PRV challenge. In summary, the recombinant virus rPRV-AD shows promise as a bivalent vaccine against both TGEV and PRV.

## 2. Materials and Methods

### 2.1. Cells, Viruses, Plasmids, and Antibodies

The swine testicular (ST), porcine kidney (PK-15), porcine intestinal epithelial (IPEC-J2), and African green monkey kidney (Vero) cell lines were purchased from Univ Biotechnology Co., Ltd. (Shanghai, China). Cell lines were authenticated by the Cellosaurus database: ST (RRID: CVCL_2329), PK-15 (R Cell lines were authenticated by RID: CVCL_2160), IPEC-J2 (RRID: CVCL_2246), Vero (RRID: CVCL_0059). All cell lines were preserved and cultured in Henan Provincial Key Laboratory of Animal Food Safety. All cells were cultured in Dulbecco’s modified Eagle’s medium (DMEM) supplemented with 8% fetal bovine serum (FBS), 100 U/mL penicillin, and 100 μg/mL streptomycin at 37 °C in a humidified incubator containing 5% CO_2_. All cell batches were routinely tested for mycoplasma contamination prior to use.

The triple-gene-deleted strain rPRV NY-gE^−^/gI^−^/TK^−^ was previously generated by deleting the *TK*, *gE*, and *gI* genes of the virulent PRV NY variant strain (GenBank accession no. KF130883), which was originally isolated from a piglet exhibiting neurological signs in Henan Province of China in 2012. This attenuated strain served as the vector backbone for constructing rPRV-AD, while the parental PRV NY strain was used as the challenge virus in piglets [24]. The PRV transfer plasmid pG-EGFP and the CRISPR/Cas9 plasmid pX459-gRNA1-EZ-gRNA2 (the latter used to excise the EGFP expression cassette) have been described previously [24]. The TGEV KF strain (a virulent field isolate from Kaifeng, Henan) was propagated in ST cells to a titer of 10^5.0^ TCID _50_/mL and used for neutralization assays; the virus was stored at the Henan Provincial Key Laboratory of Animal Food Safety.

### 2.2. Construction of the Recombinant Transfer Plasmid

The recombinant transfer plasmid pG-AD-EGFP was constructed as described previously [25]. Briefly, a pair of specific primers (AD-F: 5′-CGGATCC**CT**ATGGTCACTCTTGATATCTCATG-3′ and AD-R: 5′-CGGATCCTTAAACAGCTGTGGCATCTAAAA-3′) was designed to amplify the AD fragment containing the major neutralizing epitopes A and D according to the TGEV S gene sequence (GenBank accession: DQ81178.1), and *Bam*HI restriction site was underlined at the 5′ terminus of each primer. The AD fragment was amplified using KOD One™ PCR Master Mix (Toyobo, Shanghai, China). The PCR product was digested with *Bam*HI and ligated into the *Bam*HI-linearized, alkaline phosphatase-treated PRV transfer plasmid pG-EGFP. The AD fragment was designed to be inserted downstream of the gG promoter within the gG locus (between pK and gD) of the PRV genome. The recombinant plasmid pG-AD-EGFP was purified using the End-free Plasmid Mini Kit II (Omega Bio-Tek, Inc., Norwalk, CT, USA) according to the manufacturer’s instructions and further verified by restriction digestion and Sanger sequencing (Tsingke Biotechnology, Beijing, China).

### 2.3. Construction of the Recombinant Virus rPRV-AD

ST cells grown to approximately 80% confluence in six-well plates were infected with a 10^−4^ dilution of the parental rPRV NY-gE−/gI−/TK− strain. After 2 h of infection, the cells were transfected with the transfer plasmid pG-AD-EGFP using ExFect Transfection Reagent (Vazyme Biotech Co., Ltd., Nanjing, China). The cells were harvested when approximately 80% cytopathic effects (CPE) were observed, and the intermediate recombinant virus rPRV-AD-EGFP was purified by screening the green, fluorescent plaque and identified by PCR amplification and sequencing of the AD fragment. Recombinant rPRV-AD was obtained by plaque purification.

We inoculated purified rPRV-AD-EGFP onto ST cells pre-transfected with the CRISPR/Cas9 plasmid pX459-gRNA1-EZ-gRNA2. After three rounds of plaque purification to select non-green-fluorescent plaques as previously described [24], five non-fluorescent plaques were picked and propagated individually. Viral genomic DNA was extracted from 200 μL of each supernatant using the TaKaRa MiniBEST Viral RNA/DNA Extraction Kit Ver.5.0 (Takara Bio Inc., Dalian, China) and then amplified by PCR with primer sets AD-F/R and EGFP-F/R (F: 5′-GATTCTGTGGATAACCGTAT-3′ and R: 5′-ACAA TTTTACGCCTTAAAG AT-3′). The PCR products were sequenced, and the results confirmed that the AD fragment was retained while EGFP was deleted; the resulting recombinant virus was designated rPRV-AD.

### 2.4. Transcription and Expression of the AD Protein in the Recombinant Virus

Total RNA was extracted from the ST cells infected with rPRV-AD using the HP Total RNA Kit (Beijing Yunteide Biotechnology Co., Ltd., Beijing, China), and cDNA was then acquired by reverse transcription using the HiScript^®^ II 1st Strand cDNA Synthesis Kit (Vazyme Biotech Co., Ltd., Nanjing, China). PCR was performed using the primer pair AD-F/R.

Expression of the TGEV AD antigen was further assessed by Western blotting and indirect immunofluorescence assay (IFA), as previously described [26] with minor modifications. For Western blot analysis, ST cells were infected with fifth-passage rPRV-AD at a multiplicity of infection (MOI) of 1, with uninfected cells serving as the negative control. At 36 h post-infection (hpi), the cells were harvested and sequentially incubated with a rabbit polyclonal antibody against the TGEV AD antigen (1:300) and horseradish peroxidase-conjugated goat anti-rabbit IgG (1:5000). The specific protein signal was visualized using a DAB substrate kit (Thermo Fisher Scientific, Waltham, MA, USA).

For IFA, ST cells were infected with fifth-passage rPRV-AD at an MOI of 0.1, with uninfected cells as the negative control. At 24 hpi, the cells were processed and incubated with the rabbit anti-AD polyclonal antibody (1:50), followed by FITC-conjugated goat anti-rabbit IgG (Proteintech Group, Inc., Wuhan, China) at a dilution of 1:5000. Fluorescence signals were observed under a Nikon Eclipse Ti-U inverted fluorescence microscope (Nikon, Tokyo, Japan).

### 2.5. Biological Characteristics of Recombinant Virus

#### 2.5.1. Replication in Different Cell Lines

The recombinant virus rPRV-AD was serially diluted 10-fold in DMEM and inoculated onto confluent monolayers of ST, PK-15, Vero, and IPEC-J2 cells in 96-well plates. Cytopathic effects (CPE) were monitored daily. At 7 days post-inoculation, viral titers for each cell line were determined by the 50% tissue culture infectious dose (TCID_50_) and calculated using the Reed–Muench method.

#### 2.5.2. One-Step Growth Curves

ST cell monolayers cultured in 12-well plates were infected in triplicate with rPRV-AD or the parental rPRV NY-gE^−^/gI^−^/TK^−^ strain at an MOI of 0.1. After virus adsorption at 37 °C for 1 h, the inoculum was discarded and replaced with DMEM containing 2% FBS and 1% penicillin–streptomycin. Cell lysates were collected at 2, 4, 6, 8, 12, 24, 36, and 48 hpi, and viral titers were quantified in ST cells by the Reed–Muench method.

#### 2.5.3. Physicochemical Properties

To evaluate viral sensitivity to physical conditions, each virus (rPRV-AD and the parental rPRV NY-gE^−^/gI^−^/TK^−^ strain) was aliquoted for each treatment with three independent biological replicates per condition, and untreated virus served as the positive control. The aliquots were exposed to UV irradiation (30 min), 0.2% formaldehyde (37 °C, 24 h), 4.8% chloroform (4 °C, 30 min), 0.5% trypsin (37 °C, 1 h), pH 3.0 or 11.0 (37 °C, 1 h), or 56 °C (30 min). After each treatment, residual infectivity in ST cells was determined by CPE observation, and TCID_50_ values were calculated using the Reed–Muench method.

### 2.6. Genetic Stability

rPRV-AD was serially passaged in ST cells for 30 passages, and the AD fragment derived from the TGEV S gene was amplified, purified, and sequenced every 5 passages using primers AD-F/R. Sequence alignment was subsequently performed to evaluate the genetic stability of the inserted TGEV S gene AD fragment in the recombinant virus.

### 2.7. Safety and Immunogenicity of rPRV-AD in Piglets

To evaluate the safety and immunogenicity of rPRV-AD, eighteen 2-week-old healthy Duroc-Landrace-Yorkshire crossbred piglets of mixed sex and similar body weights were purchased. All tested negative for PRV, PEDV, TGEV, PDCoV, and porcine rotavirus by PCR or RT-PCR from rectal swabs. After a 3-day acclimation, piglets were stratified by body weight and sex and randomly assigned using a computer-generated sequence to two experimental sets (*n* = 9 each), each comprising three treatment subgroups (*n* = 3 each). Piglets from different groups were housed in separate isolation rooms to prevent cross-contamination. Daily clinical observations were performed by two trained investigators blinded to group assignments; any disagreements were resolved by consensus.

Set 1 was used exclusively for TGEV immunization and challenge. Piglets were intramuscularly immunized with 2 mL of rPRV-AD (viral dose: 10^6^ TCID_50_/0.1 mL), 2 mL of a commercial inactivated TGEV–PEDV vaccine (Wuhan Keqian Biology Co., Ltd., Wuhan, China), or 2 mL of DMEM. Set 2 was used only for PRV immunization and challenge. Piglets received the same dose of rPRV-AD (2 mL), 2 mL of a commercial Bartha-K61 live-attenuated PRV vaccine (Harbin Pharmaceutical Group Bio-vaccine Co., Ltd., Harbin, China), or 2 mL of DMEM via intramuscular injection. Rectal temperature, feed intake, and clinical signs were monitored daily. Blood samples were collected at 7, 14, 21, and 28 days post-immunization (DPI, pre-challenge), and separated sera were stored at −80 °C until analysis. Virus challenge was performed at 28 DPI. Additional serum samples were obtained at 35 and 42 DPI (post-challenge). Antibody levels at 35 and 42 DPI are influenced by both vaccination and challenge-virus-induced immune stimulation.

#### 2.7.1. Enzyme-Linked Immunosorbent Assay (ELISA)

TGEV-specific serum antibodies in Set 1 were measured using the INgezim^®^ TGEV 2.0 Blocking ELISA Kit(INGENASA-Gold Standard Diagnostics, Madrid, Spain), whereas PRV-specific antibodies in Set 2 were detected using the INgezim^®^ PRV Compac Blocking ELISA Kit (Ingenasa, Madrid, Spain). All assays were performed according to the manufacturer’s instructions.

#### 2.7.2. Neutralizing Antibody Assay

Serum samples were heat-inactivated at 56 °C for 30 min and then serially twofold diluted in DMEM. Each dilution was mixed with an equal volume of the corresponding virus suspension containing 200 TCID_50_ and incubated at 37 °C for 1 h. The mixtures were subsequently added to ST cell monolayers in 96-well plates, and CPE was monitored for 5–7 days. Neutralizing antibody titers were defined as the reciprocal of the highest serum dilution that prevented CPE in 50% of inoculated wells and were calculated using the Reed–Muench method.

#### 2.7.3. Challenge Experiments

At 4 weeks post-immunization, piglets in Set 1 received an oral challenge with 4 mL of virulent TGEV KF strain (10^5^·^0^ TCID_50_/mL), while those in Set 2 were challenged intramuscularly with 1 mL of PRV NY strain at the same titer. After challenge, we recorded rectal temperature, feed intake, clinical signs, and mortality daily for all piglets throughout the observation period. The humane endpoints were predefined as severe lethargy, persistent anorexia, abnormal body temperature, severe weight loss, or ambulatory impairment. Any piglet meeting these criteria was immediately euthanized. Animals that died unexpectedly or were euthanized were necropsied immediately. Tissues were collected according to these predefined humane endpoints. In the TGEV group, one control piglet died at 33 DPI (5 dpc) and was sampled at that time, while the remaining survivors were sampled at the scheduled terminal point. In the PRV group, two of the three DMEM-control piglets died before 42 DPI and underwent immediate tissue collection. The third survivor was euthanized and sampled at 42 DPI. All other surviving piglets in both experimental groups were euthanized and sampled at 42 DPI. Given that viral loads are time-dependent and sampling times differed between non-survivors and survivors, we did not perform formal group-wise statistical comparisons for tissue viral load data. Instead, individual measurements are presented with their corresponding sampling times, and the data are interpreted descriptively. TGEV loads in intestinal tissues, and fecal samples were quantified by RT-qPCR as previously described [27]. PRV loads in the heart, liver, spleen, lung, kidney, brain, and lymph nodes were detected by qPCR [28]. Protective efficacy was evaluated based on survival rates, clinical symptoms, and tissue viral loads. The detailed animal immunization–challenge experimental design is summarized in Supplementary Table S1.

### 2.8. Statistical Analysis

Data are shown as mean ± SD. Because of the small group size (*n* = 3), repeated-measures mixed-effects models were not feasible. For serological data, we performed cross-sectional comparisons at each time point separately, using one-way ANOVA followed by Tukey’s multiple-comparison test for pairwise group differences; no additional correction was applied across time points to avoid over-penalization, and *p* < 0.05 was considered significant for each cross-sectional analysis. For tissue viral loads, sampling times differed between early-death and endpoint animals; we therefore restricted comparisons to group differences within the same sampling time (e.g., early-death vs. early-death, or 14-dpc endpoint vs. 14-dpc endpoint) and did not perform longitudinal comparisons across different time points. All analyses were performed using GraphPad Prism 9.0.

During the preparation of this manuscript, the generative-AI tools Doubao and DeepSeek were used for English language polishing and text reorganization. All AI-generated outputs were fully reviewed and revised by the authors, and the authors take full responsibility for all content of this work.

## 3. Results

### 3.1. Generation of the Recombinant Virus rPRV-AD

**Figure 1:**
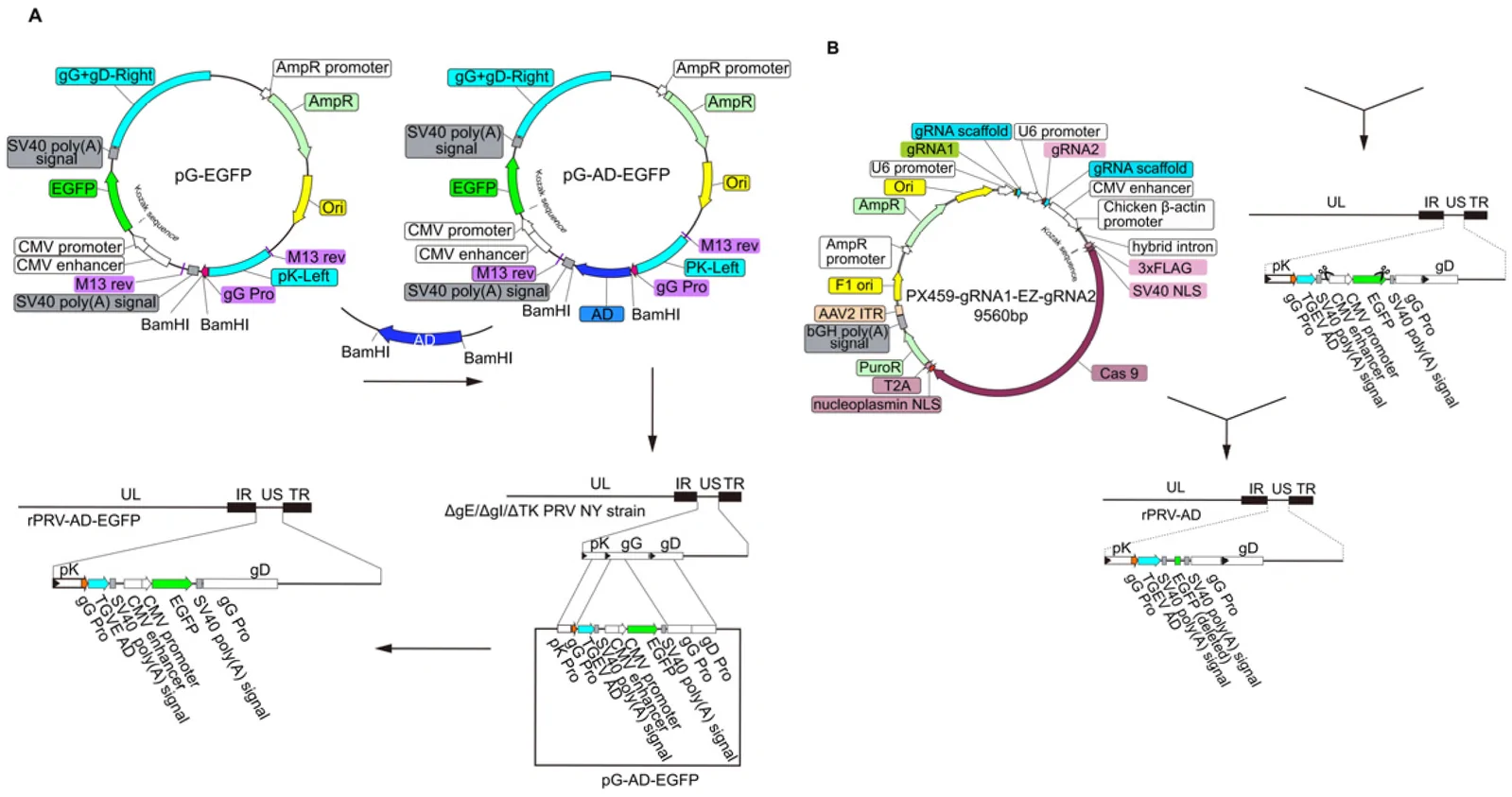
Construction flow chart of recombinant virus rPRV-AD. (**A**) Construction of transfer plasmid pG-AD-EGFP and generation of intermediate rPRV-AD-EGFP via homologous recombination. (**B**) Deletion of the EGFP reporter gene mediated by CRISPR/Cas9 system to construct the final recombinant virus rPRV-AD.

**Figure 2:**
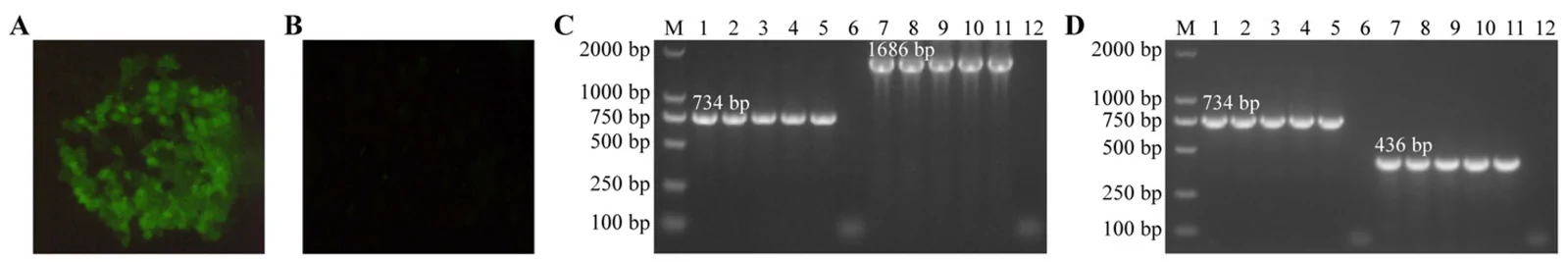
Identification of the recombinant viruses rPRV-AD. (**A**): Green fluorescent lesions of recombinant virus rPRV-AD-EGFP under fluorescence microscope. Left: Recombinant virus rPRV-AD-EGFP green plaques under blue light; Right: Parental strain plaques under blue light; (**B**): PCR identification for recombinant virus rPRV-AD-EGFP. (**C**): PCR identification for recombinant virus rPRV-AD. M: DL2000 DNA Marker; 1–5: AD gene, 734 bp; 7–11: EGFP expression box,1686 bp; 6 and 12: Negative control. (**D**): PCR identification for recombinant virus rPRV-AD. M: DL2000 DNA Marker; 1–5: AD gene, 734 bp; 7–11: Deleted EGFP fragment, 436 bp; 6 and 12: Negative control.

The recombinant virus rPRV-AD expressing the AD fragment of the TGEV S gene was constructed (Figure 1). Firstly, the AD fragment was amplified and cloned into the *Bam*H I site of PRV transfer plasmid pG-EGFP. The resulting transfer plasmid pG-AD-EGFP contained the expression cassette “gG LHA–AD–SV40 poly(A)–CMV–EGFP–SV40 poly(A)–gG RHA” and was transfected into ST cells infected with the PRV NY-gE^-^/gI^-^/TK^-^ strain. Distinct green fluorescence was observed in infected cells (Figure 2A), and the intermediate recombinant virus rPRV-AD-EGFP was purified by screening for green, fluorescent plaques over three rounds. The AD fragment (approximately 734 bp) was detected in the viral genome by PCR (Figure 2C), confirming the successful rescue of rPRV-AD-EGFP.

Subsequently, the recombinant virus rPRV-AD was rescued by inoculating purified rPRV-AD-EGFP into ST cells pre-transfected with the plasmid PX459-gRNA1-EZ-gRNA2, followed by three rounds of non-fluorescent plaque purification. PCR detection using EGFP-specific primers EGFP-F/R yielded an amplified EGFP fragment of approximately 436 bp (Figure 2D), which was significantly shorter than the expected full-length fragment (1668 bp) (Figure 2C). Sequencing further confirmed that the 1232 bp EGFP expression cassette was successfully knocked out from the rPRV-AD genome. Collectively, these results indicated that the TGEV AD fragment was correctly inserted into the PRV genome via homologous recombination, and the EGFP cassette was precisely deleted using CRISPR/Cas9 gene editing technology.

### 3.2. Expression of the Recombinant Virus rPRV-AD

**Figure 3:**
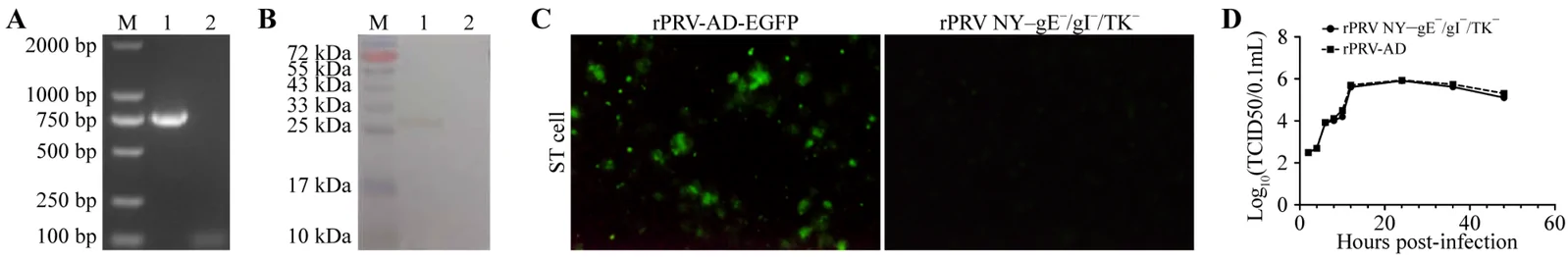
Expression of the Recombinant Virus rPRV-AD. (**A**): The PCR amplification results of TGEV AD gene; M: DL2000 DNA Marker; 1. AD gene, 734 bp; 2. Negative control. (**B**): Identification of AD protein of rPRV-AD by Western blot; M: Protein marker; 1: AD protein of rPRV-AD, 29.7 kDa. 2: Parental strain (rPRV NY–gE^−^/gI^−^/TK^−^) control. (**C**): Indirect immunofluorescence identification of AD protein of rPRV-AD strain (20×); Left: rPRV-AD strain; Right: Parental strain (rPRV NY–gE^−^/gI^−^/TK^−^) control. (**D**): One-step growth curve of recombinant virus rPRV-AD, and parental strain rPRV NY–gE^−^/gI^−^/TK^−^ on ST cells. In Figures (**B**,**C**), uninfected ST cells served as the blank negative control, and parental-PRV-infected ST cells were used as the parental-virus infection control.

Transcription and expression of the TGEV AD antigen were further verified by RT-PCR, Western blotting, and IFA. RT-PCR yielded the expected 734 bp AD-specific product (Figure 3A), and sequencing confirmed that the nucleotide sequence was identical to the reference TGEV AD sequence. Western blotting revealed a specific protein band of approximately 29.7 kDa in rPRV-AD-infected ST cells, while no corresponding band was observed in cells infected with the parental rPRV NY–gE^−^/gI^−^/TK^−^ strain (Figure 3B). The uncropped original Western blot image is provided in Supplementary Figure S1. Consistently, IFA detected specific green fluorescence in rPRV-AD-infected cells, whereas no fluorescence was observed in the parental-virus-infected controls (Figure 3C). These results demonstrate the successful rescue of recombinant rPRV-AD and stable expression of the TGEV AD antigen in infected ST cells. The nucleotide and deduced amino-acid sequences of the AD fragment are provided in Supplementary Table S4.

### 3.3. Biological Characteristics of the Recombinant Virus rPRV-AD

The growth characteristics of rPRV-AD were compared with those of the parental rPRV NY-gE^−^/gI^−^/TK^−^ strain to determine whether insertion of the TGEV AD fragment affected viral replication. Both viruses replicated efficiently in ST, PK-15, Vero, and IPEC-J2 cells, with the highest titers observed in ST cells. In infected ST cells, mild cytopathic effects, including cell rounding and shrinkage, appeared at 6 hpi, followed by partial cell detachment at 12 hpi and extensive CPE at 24 hpi. One-step growth curves revealed similar replication kinetics between the two viruses, with viral titers increasing from 6 to 36 hpi and peaking at 24 hpi (Figure 3D). The peak titer of rPRV-AD exceeded 6 log_10_ TCID_50_/mL, and no significant differences were observed at any time point (*p* > 0.05). These results indicated that insertion of the TGEV AD fragment did not significantly alter the in vitro replication properties of the parental PRV vector. The physicochemical properties of rPRV-AD were also comparable to those of the parental strain. Both viruses were completely inactivated by 0.2% formaldehyde, heating at 37 °C for 24 h, 0.5% trypsin at 37 °C for 1 h or exposure to pH 3.0 or pH 11.0. Treatment with 4.8% chloroform at 4 °C for 30 min or heating at 56 °C for 30 min markedly reduced viral infectivity, with residual titers of 10^0.75^ TCID50/mL for rPRV-AD and 10^0.875^ TCID_50_/mL for the parental strain. In contrast, UV irradiation for 30 min had a relatively limited effect, with residual titers of 10^5.535^ and 10^5.355^ TCID_50_/mL, respectively. Taken together, the insertion of the TGEV AD fragment did not substantially alter the physicochemical properties of the parental virus. The physical-chemical properties of recombinant rPRV-AD virus are provided in Supplementary Table S2.

### 3.4. Genetic Stability and Safety of the Recombinant Virus in Piglets

**Figure 4:**
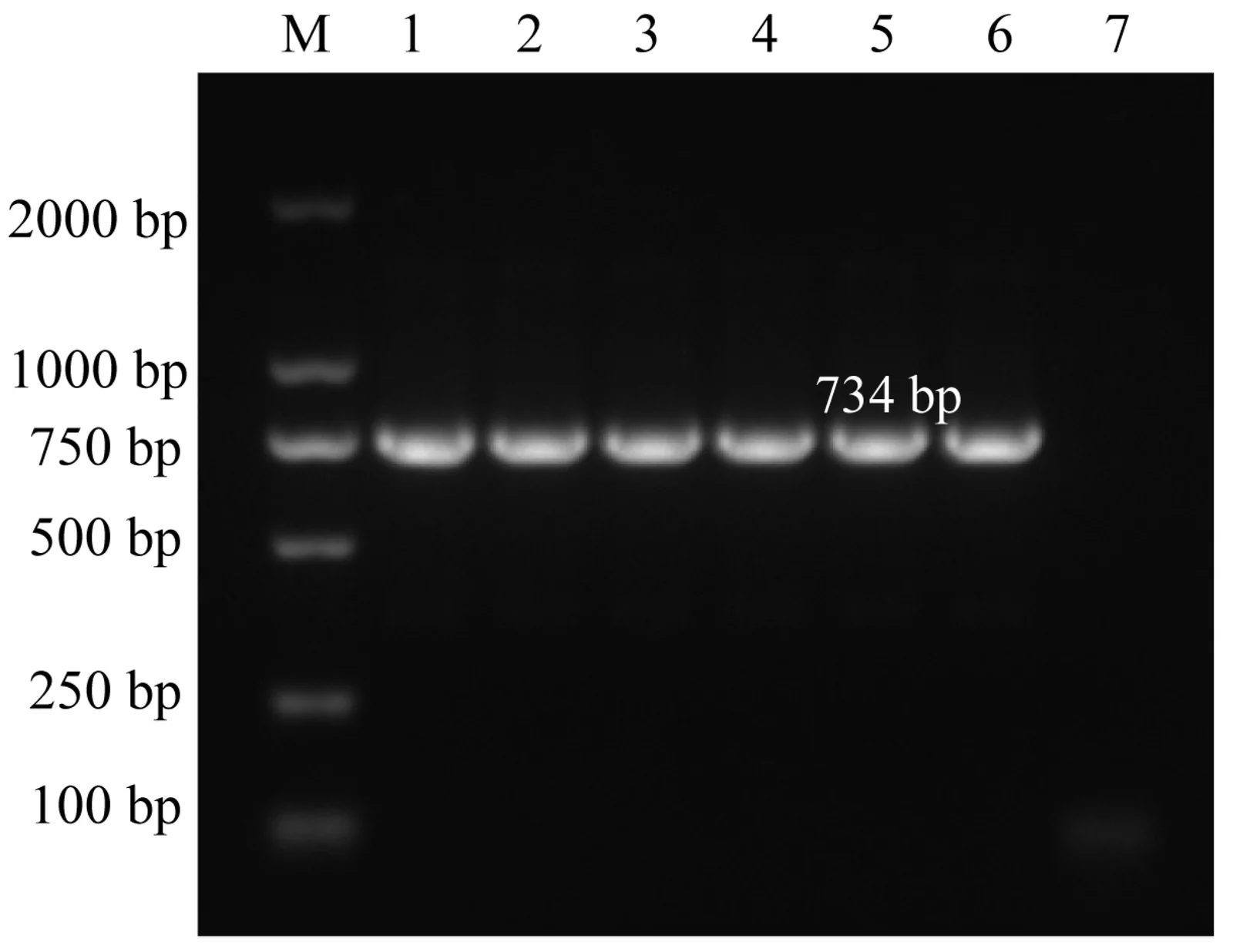
Genetic stability of the AD expression cassette in rPRV-AD. M: DL2000 DNA marker; Lanes 1–6: PCR amplification products of the AD fragment from viral DNA of the 5th, 10th, 15th, 20th, 25th, and 30th passages recombinant viruses, respectively; Lane 7: Negative control.

rPRV-AD was serially passaged in ST cells for 30 passages, and the TGEV S gene AD fragment was amplified every five passages. The AD fragment was successfully amplified from viral genomic DNA of the 5th, 10th, 15th, 20th, 25th, and 30th passages of rPRV-AD (Figure 4). Sequence analysis confirmed no mutations or deletions within the chimeric TGEV AD expression cassette, demonstrating that the exogenous AD fragment was stably retained and faithfully transmitted in progeny viruses.

Piglets immunized with rPRV-AD exhibited normal mental status, feed intake, and rectal temperature throughout the observation period, with no mortality observed. Under the conditions of this preliminary animal experiment, no obvious adverse clinical reactions were observed in the three piglets immunized with rPRV-AD.

### 3.5. Specific Antibody Response Induced by the Recombinant Virus in Piglets

**Figure 5:**
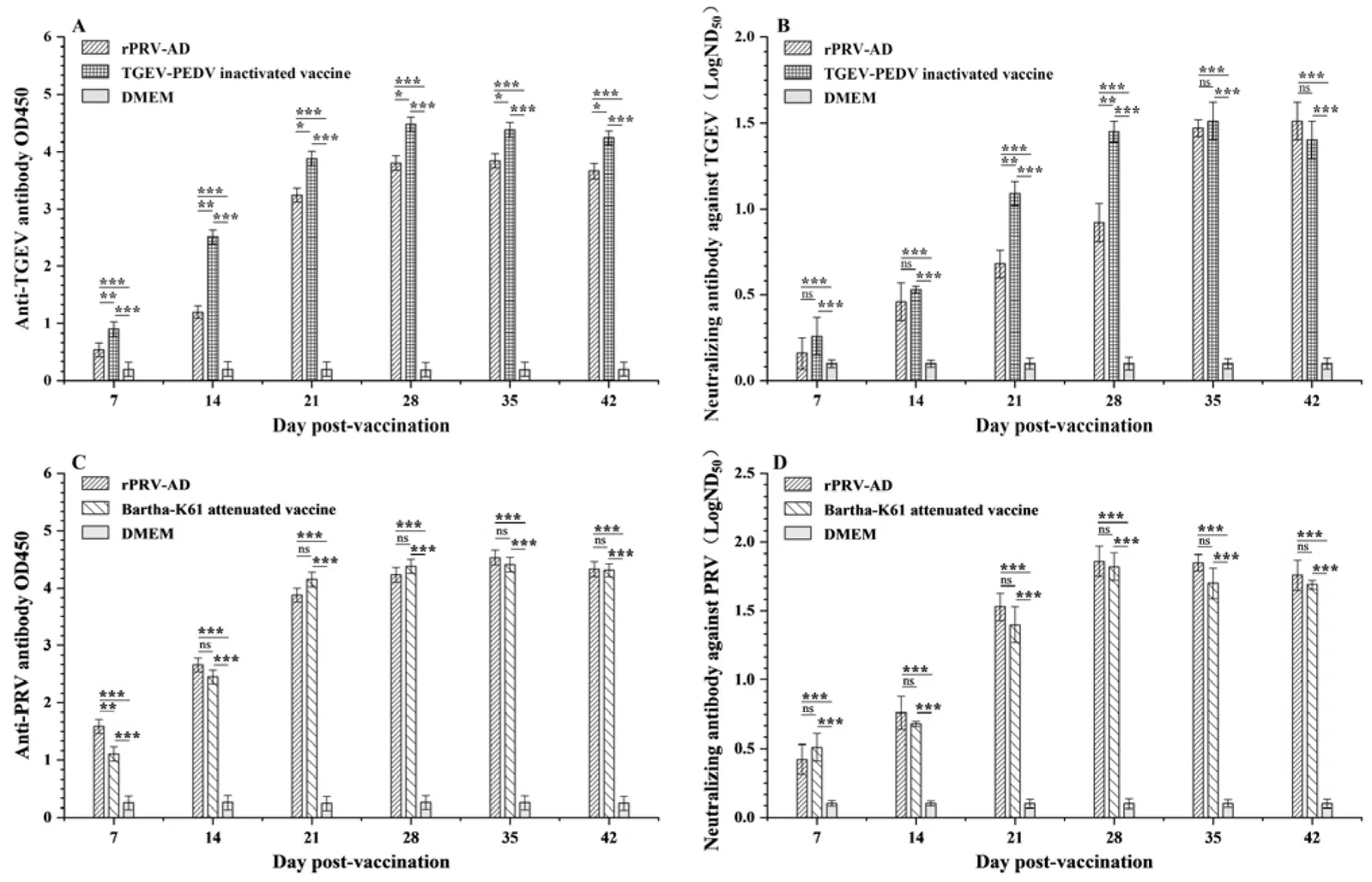
TGEV- and PRV-specific antibody responses in immunized piglets. Sera were collected at 7, 14, 21, 28, 35, and 42 DPI (pre-challenge for 7–28 DPI; post-challenge for 35–42 DPI). Antibody levels at 35 and 42 DPI reflect combined effects of vaccination and challenge infection. (**A**) TGEV-specific ELISA antibody levels. (**B**) TGEV-specific neutralizing antibody titers. (**C**) PRV gB-specific ELISA antibody levels. (**D**) PRV-specific neutralizing antibody titers. Error bars denote SD. For TGEV-specific responses, significant differences between the rPRV-AD and commercial vaccine groups were observed in ELISA levels at 7 DPI (*p* < 0.01) and in neutralizing titers at 7 DPI (*p* < 0.001). Statistical analysis was performed by one-way ANOVA with Tukey’s post-hoc test. *, *p* < 0.05; **, *p* < 0.01; ***, *p* < 0.001.

In Set 1, neither TGEV-specific ELISA antibodies nor neutralizing antibodies were detectable in the DMEM control group throughout the 4-week observation period. Both the rPRV-AD group and the commercial TGEV-PEDV inactivated vaccine group generated clear TGEV-specific antibody responses at 7 DPI (Figure 5A,B). TGEV-specific antibody levels increased rapidly from 14 to 21 DPI in both vaccine groups and continued to rise slowly after 28 DPI. Significant differences in ELISA antibody levels were observed between the two vaccine groups at 7 DPI (*p* < 0.01) (Figure 5A), while neutralizing antibody titers differed significantly as early as 7 DPI (*p* < 0.001) (Figure 5B). Overall, ELISA and neutralizing antibody assays showed similar kinetic trends for TGEV-specific antibodies in the two groups. These results indicate that the commercial TGEV-PEDV inactivated vaccine induced higher overall TGEV-specific antibody levels than rPRV-AD.

In Set 2, neither PRV-specific ELISA antibodies nor neutralizing antibodies were detectable in the DMEM control group. Both the rPRV-AD and Bartha-K61 groups generated PRV gB-specific serum antibodies by 7 DPI (Figure 5C,D). Although ELISA antibody levels induced by rPRV-AD were lower than those elicited by Bartha-K61, rPRV-AD still produced readily detectable PRV-neutralizing antibodies. Neutralizing antibody titers differed significantly between the two vaccine groups at all time points tested (*p* < 0.001). Together with the TGEV-specific responses observed in Set 1, these results show that rPRV-AD induces both binding and neutralizing antibodies against TGEV and PRV, supporting its potential as a bivalent vaccine candidate.

### 3.6. Protection of Piglets Against the Virulent Challenge of TGEV

At 4 weeks post-immunization, piglets in Set 1 were orally challenged with 4 mL of the virulent TGEV KF strain at 10^5^·^0^ TCID_50_/mL. In the rPRV-AD group, two of three piglets developed transient mild diarrhea within 36 hpc and subsequently recovered; the third remained asymptomatic. Piglets immunized with the commercial TGEV-PEDV inactivated vaccine remained clinically healthy throughout the observation period. In contrast, all DMEM-treated piglets showed signs of lethargy, anorexia, and diarrhea. One control piglet developed severe watery diarrhea, vomiting, and dehydration at 3 dpc and died at 5 dpc; the other two recovered gradually.

Given the discrepancy in sampling time points between animals that died early at 5 days post-challenge (dpc) and surviving animals euthanized at the experimental endpoint of 14 dpc, all individual viral load data were analyzed and presented in a purely descriptive manner. Piglets immunized with rPRV-AD or the commercial vaccine had lower viral loads in intestinal tissues and feces than DMEM-treated controls, with the commercial vaccine showing the best clearance. Tissue and fecal samples were collected from pigs at death or euthanasia, and TGEV loads were measured by RT-qPCR and expressed as log_10_ copies per gram; individual values are listed in Supplementary Table S3. The commercial vaccine group exhibited the lowest titers in both tissues and feces, with only slight differences between sample types. These observations are cross-sectional per time point, as no longitudinal comparisons were made. Overall, rPRV-AD appeared to reduce clinical signs and viral replication upon challenge, though its efficacy was somewhat lower than that of the commercial TGEV–PEDV inactivated vaccine based on these descriptive data.

### 3.7. Protection of Piglets Against the Virulent Challenge of PRV

At 4 weeks post-immunization, piglets in Set 2 were intramuscularly challenged with 1 mL of the virulent PRV NY strain at 10^5^·^0^ TCID_50_/mL. All rPRV-AD-immunized piglets remained clinically healthy throughout the observation period. In the Bartha-K61 group, one piglet developed mild neurological signs at 120 hpc but recovered spontaneously; the other two showed no clinical abnormalities. In the DMEM control group, two of three piglets developed neurological dysfunction, diarrhea, and vomiting at 96 hpc and died (one at 4 dpc and the other at 6 dpc); the third survivor was euthanized at the scheduled endpoint (14 dpc).

**Figure 6:**
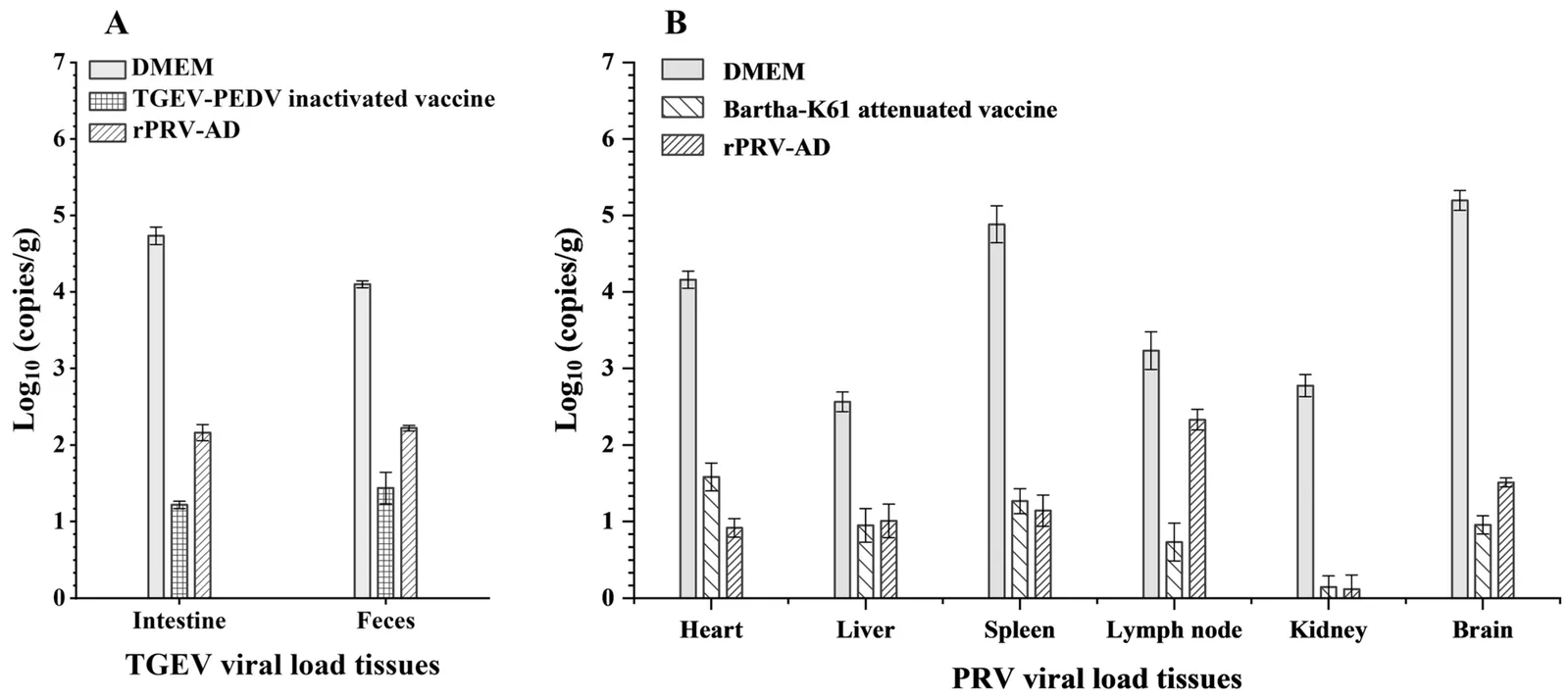
Quantification of viral loads in tissue and fecal samples following virulent challenge. (**A**) TGEV RNA loads in intestinal tissues and feces (log_10_ copies/g). (**B**) PRV DNA loads in heart, liver, spleen, lymph node, kidney, and brain (log_10_ copies/g). Data are presented as individual values with means ± SD. Due to unequal sampling times between early-death and endpoint animals (as detailed in Section 2.7.3), statistical comparisons were not performed across different time points; instead, only descriptive data are shown.

PRV loads in various tissues were quantified by qPCR from piglets that died early or were euthanized at 14 dpc. In the DMEM group, the brain consistently showed the highest viral loads (individual values ranging from 10^6^ to 10^8^ copies/g), followed by the spleen and heart, while kidney loads were the lowest across all three animals (Figure 6B). In the Bartha-K61 group, all three piglets survived to 14 dpc, with tissue loads ranging from 10^2^ to 10^4^ copies/g across different organs. In the rPRV-AD group, all three piglets also survived to 14 dpc, with loads falling within a similar range as the Bartha-K61 group, except for somewhat higher values observed in the brain and lymph nodes of two individuals (Figure 6B). These observations, with individual sampling times indicated in the figure legend, are presented descriptively; no formal group-wise statistical comparisons were performed for tissue viral load data due to unequal sampling times between early-death and endpoint animals.

## 4. Discussion

Conventional commercial vaccines help control TGEV infections, but continuous antigenic drift and genetic recombination among circulating strains gradually erode their protective efficacy, leading to repeated outbreaks in swine herds [29,30]. Conventional vaccines mainly induce humoral immunity, whereas TGEV invades and replicates primarily in mucosal tissues. In addition, mutations in key neutralizing epitopes enable immune escape. This combination of mucosal infection and systemic immunization often results in breakthrough infections even in vaccinated pigs [31,32,33]. Several newer vaccine platforms—including mRNA vaccines, virus-like particle vaccines, and probiotic-based oral vaccines—still face practical obstacles such as strict cold-chain requirements, complex purification processes, and poor antigen stability in vivo [34,35,36,37,38]. In this study, we selected the highly conserved A and D antigenic sites of the TGEV S protein for recombinant vaccine construction. This approach may reduce the risk of vaccine failure due to viral variation and offers a potentially useful strategy for developing a broadly protective TGEV vaccine.

Since the 2011 variant PRV outbreak in China, the Bartha-K61 vaccine has not provided adequate protection against circulating field strains, and pseudorabies remains sporadic in intensive pig farms [9,10,39]. PRV and TGEV are both widespread in large-scale pig operations, so a bivalent vaccine is clearly needed. Recombinant live-attenuated PRV-vectored vaccines have attracted considerable interest [40,41,42,43,44,45]. The PRV gG gene encodes a non-essential glycoprotein with strong endogenous promoter activity, which drives exogenous gene transcription [15,46]. Deleting gG does not impair viral growth, making it a suitable insertion site [47]. Here, the left homologous arm of the transfer plasmid pG contained the gG promoter region, which we used to drive expression of the inserted gene; the exogenous fragment was cloned into the BamHI site, generating the recombinant plasmid pG-AD-EGFP. Following homologous recombination with the PRV NY-gE^−^/gI^−^/TK^−^ genome, rPRV-AD-EGFP was obtained [47], the viral DNA is prone to fragmentation during extraction, which severely limits the co-transfection efficiency of the recombinant plasmid and the viral genome. To overcome this, we optimized the transfection protocol by first inoculating ST cells with the virus, followed by transfection. The results demonstrated that this modified procedure is simple and time-saving, without significantly affecting transfection efficiency.

The tandem A and D epitope fragments were derived from the TGEV S glycoprotein [4,48,49]. Both epitopes have low mutation frequencies in field strains and were linked into a single expression cassette to reduce the chance of immune escape [8]. Epitope A requires eukaryotic glycosylation for its native conformation and neutralizing antibody induction; epitope D complements A by blocking virion infectivity and limiting tissue spread [50]. In rPRV-AD-infected ST cells, the recombinant AD protein was processed and detected as a 29.7 kDa band by Western blotting, confirming its stable expression. This provides the molecular basis for the neutralizing antibody responses observed in piglets. Although the tandem AD cassette was designed based on the naturally low mutation frequencies of these epitopes [8], this construct uses a short epitope fragment rather than the full-length S protein of TGEV. Under large-scale vaccination pressure, single-point mutations within the AD region could alter antibody binding and produce escape variants that reduce protective efficacy. Thus, field surveillance of AD epitope sequence variation will be important for future application of this vaccine candidate.

In vitro characterization showed that rPRV-AD and its parental triple-gene-deleted strain rPRV NY-gE^−^/gI^−^/TK^−^ had highly similar phenotypic profiles. One-step growth curve analysis revealed nearly identical replication kinetics in ST cells, with no significant differences in the 50% tissue culture infective dose (TCID_50_) titers at any of the time points tested (*p* > 0.05). Quantification of TCID_50_ across several mammalian cell lines confirmed that ST cells supported the highest titers for both viruses, indicating that insertion of the AD cassette did not alter the cell tropism of the pseudorabies virus (PRV) vector. Physicochemical stability assays showed that the recombinant and parental viruses had comparable resistance to heat inactivation, formaldehyde, trypsin, acidic and alkaline pH, and ultraviolet irradiation. Serial passaging in vitro verified that the TGEV AD cassette did not impair PRV replication or proliferation, and rPRV-AD remained genetically stable during continuous passage in cell culture. Under the conditions of this preliminary animal study, no mortality or obvious clinical abnormalities were observed following rPRV-AD inoculation in piglets.

Descriptive observation showed that rPRV-AD vaccination reduced viral accumulation in intestinal and fecal samples after TGEV challenge compared with the DMEM control group, although the protective effect was inferior to that of the commercial inactivated vaccine.

An ideal vaccine candidate should possess both safety and protective efficacy [15]. Here, we evaluated the immunogenicity and protective capacity of rPRV-AD against PRV and TGEV by measuring serum antibody levels (neutralization assays and indirect ELISA) in immunized piglets, and by quantifying tissue viral loads after challenge using RT-qPCR. Serological results showed that rPRV-AD induced detectable immune responses against both viruses. After TGEV challenge, intestinal viral loads correlated inversely with anti-AD antibody levels, indicating that the vaccine elicited functional TGEV-specific neutralizing antibodies. The TGEV-specific antibody response to rPRV-AD was slightly lower than that to the commercial TGEV-PEDV vaccine, but the difference in neutralizing titers was small. For PRV, rPRV-AD also induced specific binding and neutralizing antibodies; notably, its PRV antibody levels were significantly higher than those induced by Bartha-K61 (*p* < 0.05). Following PRV challenge, tissue viral loads in rPRV-AD-immunized piglets were generally comparable to those in Bartha-K61-immunized animals across most detected tissues, with only marginal numerical differences observed in lymph node viral loads between the two groups. Thus, the PRV-specific immune response elicited by rPRV-AD was roughly equivalent to that of the classical Bartha-K61 strain. Overall, rPRV-AD induced strong PRV immunity and provided moderate but measurable protection against TGEV. Compared with unvaccinated controls, rPRV-AD vaccination alleviated diarrhea and significantly reduced viral loads in both intestinal and systemic sites after TGEV challenge. In this small-scale preliminary study, rPRV-AD provided measurable dual protection against both PRV and TGEV, warranting further systematic evaluation and optimization of this novel bivalent vaccine candidate.

tissue viral loads in rPRV-AD-immunized piglets were generally comparable to those in Bartha-K61-immunized animals across most detected tissues, with only marginal numerical differences observed in lymph node viral loads between the two groups.

This study has a few limitations worth considering. The sample size was small (*n* = 3 per group), and the study was exploratory in design. These factors limit the generalizability of our statistical findings. For tissue viral loads, early-death animals and survivors were sampled at different time points. Our statistical comparisons were therefore confined to cross-sectional differences within the same sampling time, and we did not perform longitudinal comparisons across different time points. This should be kept in mind when interpreting tissue viral load data. In addition, we did not assess mucosal immune responses (e.g., intestinal sIgA) or verify the absence of viral shedding by swab sampling, even though rPRV-AD is theoretically free of such risks. We also did not measure pre-immunization baseline antibody levels; therefore, we cannot exclude the possibility that maternally derived antibodies in the 2-week-old piglets may have interfered with the results. Our study only included piglets aged 2 to 6 weeks. Given that TGEV is most lethal to piglets under 14 days of age [51], and PRV can establish long-term latent infection. Thus, it remains unclear whether our findings apply to younger pigs or reflect long-term protection. Larger sample sizes and broader age ranges will be needed to confirm these preliminary observations.

## 5. Conclusions

We observed no obvious clinical signs in vaccinated piglets. The vaccine also showed measurable protection against TGEV and PRV in this pilot study. These findings support further testing of rPRV-AD as a bivalent vaccine candidate.

## Data Availability

The original contributions presented in this study are included in the article/Supplementary Material. Further inquiries can be directed to the corresponding authors.

## Supplementary Materials

The following supporting information can be downloaded at: https://www.mdpi.com/article/10.3390/vetsci13090914/s1, Figure S1: Original uncropped Western blot images corresponding to Figure 3B; Figure S2: Figure 3B original image; Table S1: Summary of piglet immunization–challenge experimental design; Table S2: Physical and chemical characteristics of recombinant virus rPRV-AD; Table S3: Individual intestinal and fecal viral loads of piglets following TGEV challenge; Table S4: Nucleotide and deduced amino-acid sequences of the cloned TGEV AD fragment containing epitopes A and D.
