# Supplementary material for: Construction and Immunogenicity of a Recombinant Pseudorabies Virus Expressing the Major Neutralizing Epitopes A and D of Transmissible Gastroenteritis Virus Spike Protein
Source: Vet Sci. 2026 Sep 5;13(9):914. doi: 10.3390/vetsci13090914 (PMC13611863; doi:10.3390/vetsci13090914)
Supplement: Supplementary file 1 [file vetsci-13-00914-s001.zip › Supplementary Tables.pdf]

Supplementary Table S1. Summary of piglet immunization-challenge experimental design

Experimental set	Immunization group	Number of animals per	Immunization dose per piglet	Immunization route	Immunization treatment	Virus challenge (28 DPV)	Serum sampling time (DPV)	Daily monitoring items
Set1	rPRV-AD	3 piglets	2 mL	Cervical intramuscular injection	rPRV-AD (10^6 $TCID_{50}$/0.1 mL)	TGEV	7, 14, 21, 28 (pre-challenge), 35, 42	Rectal temperature, feed intake, clinical signs
	Commercial TGEV/PEDV vaccine	3 piglets	2 mL	Cervical intramuscular injection	commercial inactivated TGEV – PEDV vaccine	TGEV		
	DMEM	3 piglets	2 mL	Cervical intramuscular injection	DMEM	TGEV		
Set2	rPRV-AD	3 piglets	2 mL	Cervical intramuscular injection	rPRV-AD (10^6 $TCID_{50}$/0.1 mL)	PRV	7, 14, 21, 28 (pre-challenge), 35, 42	Rectal temperature, feed intake, clinical signs
	Commercial PRV vaccine	3 piglets	2 mL	Cervical intramuscular injection	commercial Bartha-K61 live-attenuated PRV vaccine	PRV		
	DMEM	3 piglets	2 mL	Cervical intramuscular injection	DMEM	PRV		

Supplementary Table S2 Physical and chemical characteristics of recombinant virus rPRV-AD

Treatment	rPRV-AD Test group	rPRV-AD Control group	Parental strain Test group	Parental strain Control group
Chloroform	$10^{0.75}$	10^{6}	$10^{0.875}$	10^{6}
Formaldehyde	10^{0}	10^{6}	10^{0}	10^{6}
Trypsin	10^{0}	10^{6}	10^{0}	10^{6}
Acid	10^{0}	10^{6}	10^{0}	10^{6}
Alkali	10^{0}	10^{6}	10^{0}	10^{6}
High temperature	$10^{0.5}$	10^{6}	$10^{0.5}$	10^{6}

Supplementary Table S3. Individual intestinal and fecal viral loads of piglets following TGEV challenge.

Group	Pig	Intestine (log 1_0 copies/g)	Feces (log 1_0 copies/g)	Survival Status	Sampling time (dpI)
	1	4.72	4.11	Early death	33
DMEM	2	4.88	4.26	Survived	42
	3	4.76	4.26	Survived	42
	1	1.48	2.21	Survived	42
TGEV-PEDV inactivated vaccine	2	1.32	2.22	Survived	42
	3	1.41	2.30	Survived	42
	1	1.31	2.28	Survived	42
rPRV-AD	2	1.12	2.37	Survived	42
	3	1.22	2.21	Survived	42

Note: Piglets with early death were sampled at 5 days post-challenge (dpc). Surviving piglets were euthanized and sampled at the experimental endpoint of 14 dpc. The inconsistent sampling time points resulted in unpaired individual data, which prohibits cross-individual statistical comparison of viral loads and supports only descriptive analysis in this study.

Table S4. Nucleotide and deduced amino-acid sequences of the cloned TGEV AD fragment containing epitopes A and D

Item	Sequence
Nucleotide sequence (AD fragment)	AACGGCCCGGCTCCGAGCGGTAGGACACACACACCTTTGCGCATCTCCACAGCTCAACAATGA<br>AGTGGGCAACGTGGATCC**CT**ATGGTCACTCTTGATATCTCATGTTATAAAGATACAGTGAGTG<br>ACTCGAGCTTTTCCAGTTACGGTGAAATGCCGTTCGGCGTAACTGATGGACCACGGCACTGTT<br>ACGTACTCTATAAAGGCACAGCTCTTAAGTATCTAGGAACATTACCACCTGTTGTCAAGGAGA<br>TTGCTATTAGTAAGTGGGGCCATTTTTATATTAATGGTTACAATTTCTTTAGCACATTTCCTA<br>TTGATTGTATATCTTTTAATTTGACCACTGGTGATAGTGACGTTTTCTGGACAATAGCTTACA<br>CATCGTACACTGAAGCGTTAGTACAAGTTGAAAATACAGCTATTACAAAGGTGACGTATTGTA<br>ATAGTTACGTTAATAACATTAAATGCTCTCAACTTACTGCTAATTTGAATAATGGATTTTATC<br>CTGTTTCTTCAAGTGAAGTTGGTATTGTCAATAAGAGTGTTGTGTTACTACCTAGCTTTTACA<br>CACATACCATTGTTAACATAACTATTGGTCTTGGTATGAAGCGTAGTGGTTATGGTCAACCCA<br>TAGCCTCAACATTAAGTAACATTACACTACCAATGCAGGGTGACAACACCGATGTGTACTGTA<br>TTCCTTCTGACCGATTTTCAGTTTATGTTCATTCTACTTGCAAAAGTGCTTTATGGGACAATG<br>TTTTTAAGCGAAAATGCAAGGACGTTTTAGATGCCACAGCTGTTTAAGGATCCATG
Deduced amino acid sequence	LWSLDIHVLKDTVSDSSAFQLRVKCRSAYWTHGTVTYSIKATALKYLGTLTPCVSRSLLLVSW<br>GHFYINGYQFLFSHILIVYLFNFDHW*

Note:The sequences include flanking primer-derived nucleotides and BamHI restriction enzyme recognition sites (GGATCC) used for vector construction.
