# Supplementary material for: Construction and Immunogenicity of a Recombinant Pseudorabies Virus Expressing the Major Neutralizing Epitopes A and D of Transmissible Gastroenteritis Virus Spike Protein
Source: Vet Sci. 2026 Sep 5;13(9):914. doi: 10.3390/vetsci13090914 (PMC13611863; doi:10.3390/vetsci13090914)

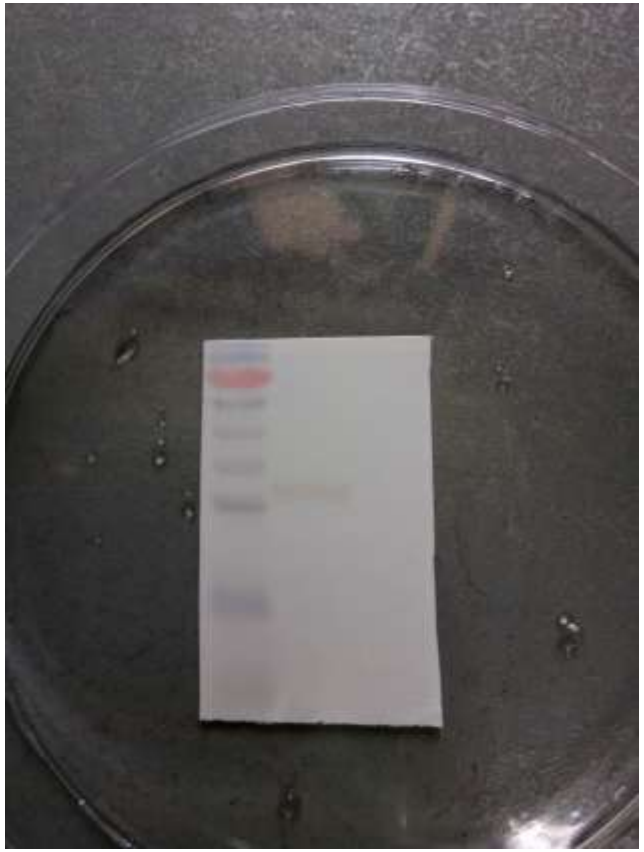

Supplement: Supplementary file 1 [file vetsci-13-00914-s001.zip › Supplementary_Figure_S2_Fig-3B original image.pdf]
